# The Current Landscape of Immune Checkpoint Inhibitor Immunotherapy for Primary and Metastatic Brain Tumors

**DOI:** 10.3390/antib12020027

**Published:** 2023-04-04

**Authors:** Paolo Alimonti, L. Nicolas Gonzalez Castro

**Affiliations:** 1 Department of Medicine, Vita-Salute San Raffaele University, Via Olgettina, 58, 20132 Milano, Italy; 2 Center for Neuro-Oncology, Dana-Farber Cancer Institute, Department of Neurology, Brigham and Women’s Hospital, Harvard Medical School, 25 Shattuck Street, Boston, MA 02115, USA

**Keywords:** immune checkpoint inhibitors, glioblastoma, IDH-mutant gliomas, meningiomas, primary CNS Lymphoma, brain metastasis

## Abstract

Antibodies against immune checkpoint inhibitors (ICIs) have revolutionized the treatment of multiple aggressive malignancies, including melanoma and non-small cell lung cancer. ICIs for the treatment of primary and metastatic brain tumors have been used with varying degrees of success. Here, we discuss the available evidence for the use of ICIs in the treatment of primary and metastatic brain tumors, highlighting challenges and opportunities for furthering this type of cancer immunotherapy in neuro-oncology.

## 1. Introduction

**Figure 1:**
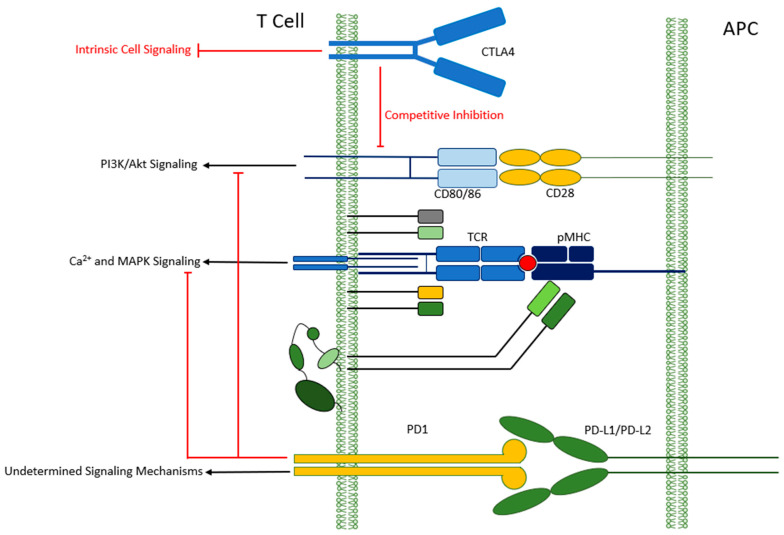
Immune checkpoint inhibition of T cell function via CTLA-4 and PD-1. CTLA-4: cytotoxic T-lymphocyte-associated protein 4. PD-1: programmed cell death protein 1. PD-L1/2: programmed death ligand 1/2. APC: Antigen presenting cell. TCR: T cell receptor. pMHC: peptide MHC complex. MHC: major histocompatibility complex.

Increased understanding of the pathways regulating the response of the immune system has led to the discovery of inhibitory pathways in lymphocytes that could be drugged to increase the activity of T lymphocytes against cancer cells, effectively heralding a new era in cancer immunotherapy. The first of these pathways to be discovered was the one modulated by the cytotoxic T-lymphocyte-associated protein 4 (CTLA-4), a surface receptor on T cells that acts to inhibit their function when bound by ligands expressed in antigen-presenting cells (APCs) [1].The second immune checkpoint inhibitor (ICI) pathway to be harnessed therapeutically is that mediated by programmed cell death protein 1 (PD-1). PD-1 is a surface receptor on activated T and B lymphocytes whose signalling enables immune tolerance after binding to PD-L1 and PD-L2 ligands expressed in APCs and tumor cells (see Figure 1) [1].

In recognition of the clinical benefit afforded by the therapeutic targeting of immune checkpoint inhibitors, the 2018 Nobel Prize in Medicine was awarded to James Allison and Tasuku Honjo for the discovery of negative immune regulation by CTLA-4 and PD-1, respectively. ICIs have been effectively deployed against multiple solid tumors, with approvals for over 20 cancer indications as of date, and a blanket indication for tumors with mismatch repair mutations [2]. Remarkable responses have been achieved for melanoma and non-small cell lung cancer (response rates of up to 60% and 100%, respectively, in some studies), where ICIs are now part of the standard of care [3,4].

Tumors of the central nervous system pose a significant therapeutic challenge for all cancer treatment modalities, with immunotherapy being no exception. In particular, the immune-privileged environment of the central nervous system (CNS) favors an immunosuppressive microenvironment with (i) a reduced number of tumor-infiltrating lymphocytes (TILs) and (ii) an overabundance of macrophages with an immunosuppressive phenotype [5]. The presence of a relatively low tumor mutational burden in most primary tumors with a limited yield of neoantigens [6] and the access limitations imposed by the blood–brain barrier [7] are additional challenges to contend with. Despite these challenging facts, multiple clinical trials evaluating the use of ICIs for the treatment of primary and metastatic brain tumors have been conducted with varying degrees of success. Here, we discuss the current data for the use of ICIs in primary brain tumors (including gliomas, meningioma, and primary central nervous system lymphoma) as well as in brain metastases (including leptomeningeal metastases).

## 2. ICI for Primary Brain Tumors

### 2.1. Glioblastoma

Glioblastoma (GBM) is the most common and malignant primary brain tumor. Despite aggressive treatment, including surgery, chemotherapy, and radiotherapy, its prognosis is still dismal, with an overall survival (OS) of 12–15 months or 9.8% in 5 years [8,9]. Disease progression is inevitable despite gross-total surgical resection due to the microscopically infiltrative nature of the tumor, which makes complete eradication impossible. Patients with progressive GBM after standard of care therapy have a median OS (mOS) of 6–11 months [10,11,12]. Given the success of ICIs in other forms of cancer and considering the several instances of PD-L1 positivity in GBM reported in the literature, several studies have investigated the use of ICIs in both primary and recurrent GBM, Table 1 [13,14,15,16].

In the KeyNote-028 study [13], 26 patients with PD-L1-positive progressive GBM received pembrolizumab every two weeks for up to 2 years. The treatment was well tolerated, but only two patients (8%) achieved the primary endpoint of objective response rate (ORR), with only partial responses. Nonetheless, the duration of response (DOR) was 8.3 and 22.8 months in these two patients, demonstrating that pembrolizumab monotherapy can induce long-lasting responses in select cases. The study enrolled a small number of patients and did not screen for the O-6-methylguanine-DNA methyl transferase (*MGMT*) promoter methylation status except in one responder (methylated *MGMT* promoter). Additionally, the PD-L1 status assessment relied on primary tumor samples, which may differ from the status at disease recurrence.

A second phase II study compared pembrolizumab and bevacizumab to pembrolizumab monotherapy in 80 patients with recurrent GBM [17]. The combination did not improve progression-free survival (PFS) and OS compared to the bevacizumab monotherapy, but the responders had a significantly longer response than those undergoing bevacizumab monotherapy. On the other hand, pembrolizumab monotherapy was ineffective, with a 0% ORR. The authors considered several reasons behind this treatment failure. First, anti-vascular endothelial growth factor (VEGF) drugs may exacerbate hypoxic conditions in the tumor microenvironment (TME) and increase the infiltration of immunosuppressive cells, contributing to tumor immunosuppression. Additionally, they noted that other monoclonal antibodies (mAbs) had reduced tumor penetration upon administration of antiangiogenic drugs [27]. In this study, the tumor immune biomarkers and immune activation gene expression profiles (GEPs) do not reliably predict therapeutic benefit in progressive GBM. Nonetheless, such conclusions rely on samples from primary GBM, and concomitant dexamethasone may have hindered or masked significant intratumoral immune activation [17].

Cloughesy et al. [24] performed mRNA expression profiling and T cell receptor (TCR) sequencing on a cohort of 32 patients receiving neoadjuvant pembrolizumab compared to adjuvant administration. Their findings suggest interferon-gamma (IFN-γ)-based activation of tumor- infiltrating lymphocytes (TILs) following neoadjuvant ICI treatment. CD4+ T cells increased their expression of activated/effector and memory markers (including CD152 and CD127), suggesting possible priming of the immune response following activation in the adjuvant setting. Importantly, T cell expansion was independent of treatment timing, but tumor-specific T cell clones retained their functionality upon subsequent administrations of the drug. This result indicates that the neoadjuvant regimen primes T cells for systemic proliferation and tumor-specific effector function. In addition, TCR diversity was not increased upon neoadjuvant PD-1 block, suggesting that T cell diversification is independent of PD-1 action. This study provided evidence in support of the neoadjuvant use of PD-1 inhibition in progressive GBM, which is yet to be validated in larger studies.

Lee et al. [28] investigated neoadjuvant pembrolizumab’s molecular and cellular effects in recurrent GBM. Specifically, they performed a single-cell analysis of the immune cells of the TME. They found that neoadjuvant PD-1 block by pembrolizumab activates T cells in the periphery and enhances their infiltration in GBM. Once inside the tumor, the CD8+ T cell population clonally expands, exerts antitumor cytotoxicity, and engages in reciprocal signaling and activation loops with conventional dendritic cells (cDCs). In turn, the activated cDCs aid T cell trafficking and infiltration via CXCL9/10/11. Neoadjuvant pembrolizumab also induces IFN-γ-related gene expression changes in monocytes and tumor-associated macrophages (TAMs). The effect on these cells is dual. On one side, it causes CXCL9/10 release to enhance T cell infiltration. Conversely, it mainly increases immunosuppression through IL-1b release and surface upregulation of immunosuppressive markers such as CXCR4, PD-L1, CTLA-4, and NECTIN2. These findings demonstrate a possible mechanism of neoadjuvant pembrolizumab’s antitumor effects, warranting additional studies with different dosing schemes [28] and drug combinations.

Several trials employed the PD-1 blocker nivolumab with both recurrent and newly diagnosed GBM. Most studies failed to improve the median OS [21,23,25,26]. One trial [20] yielded promising results with a median OS of 16.9 months in the cohort treated with combined nivolumab and the IL-12 gene therapy veledimex (VDX) at a 10 mg dose. Interestingly, the OS was down to 8.5 months in the nivolumab plus 20 mg VDX combination, suggesting that the benefit of nivolumab was dependent on a lower VDX dose. The tumor IFN-γ signature increased in post-treatment tumor samples, demonstrating the molecular effects of this ICI combination. This study confirmed that immune checkpoint signaling in GBM undergoes the first reduction after IL-12 gene therapy and another downregulation with nivolumab. Schalper et al. [23] describe the molecular effects of neoadjuvant nivolumab in both newly-diagnosed and recurrent GBM. Analyses revealed increased T cell- and IFN-γ-related gene expression and downregulated cell cycle-related gene expression in tumor cells.

An open-label phase III study compared nivolumab added to standard of care therapy (radiation and temozolomide) versus nivolumab and radiation in newly-diagnosed glioblastoma patients with an unmethylated *MGMT* promoter [29]. Overall, the mOS was similar in both arms. Temozolomide (TMZ) was responsible for chronic lymphopenia, as noted by the 10.2% vs. 1.5% incidence in grade 3/4 lymphopenia cases in nivolumab + temozolomide + radiation vs nivolumab + radiation.

The data from these studies provide evidence that nivolumab, either in monotherapy or added to standard of care therapies, does not induce a significant clinical benefit for patients with newly-diagnosed or progressive GBM. Several studies have noted the confounding effect of concurrent dexamethasone use and its suppressive effect on the mounting antitumor immune response, a fact to consider when designing eligibility criteria for future ICI studies.

Two additional studies evaluated inhibiting PD-L1 instead of PD-1, with the PD-L1 inhibitors durvalumab [18] and atezolizumab [19]. In the former study, durvalumab did not improve OS or PFS, either alone or in combination with bevacizumab. In the subset of patients noted to benefit, durvalumab induced an increase in CD8+ T cell proliferation, peaking at 15 days post-initiation of the protocol. Instead, in the study by Lukas et al. [19], atezolizumab induced one partial response and three stable responses in a 16-patient cohort with progressive GBM. Notably, one patient with isocitrate dehydrogenase (IDH)-mutant GBM experienced a greater than 16-month survival, and CD4+ T cell count positively correlated with survival; however, according to the 2021 WHO classification of central nervous system tumors, the presence of an IDH mutation is not in line with the current definition of GBM.

Finally, Omuro et al. [22] explored dual ICI with ipilimumab and nivolumab in a cohort of 40 patients with recurrent GBM. Three patients achieved partial responses, and eight demonstrated stable disease. Although the combination treatment was deemed safe overall, it was less well tolerated (higher incidence of adverse events) than nivolumab monotherapy, with tolerability limited by the dosage of ipilimumab.

Overall, although ICIs have not led to a breakthrough in the treatment of glioblastoma, there is ongoing work to understand how these therapies help modulate the TME and how they might come to play a role as part of therapeutic combinations. ICIs induce changes in gene expression and cytokine signature, particularly IFN-γ, which enables the interplay between T cells and DCs in the antitumor response [28]. ICIs also appear to induce gene expression changes in tumor cells [24], a finding that requires further characterization. Overall, IFN-γ may serve as a new biomarker to predict GBM’s response to ICIs. Moreover, the neoadjuvant administration of a PD-1 inhibitor seems to prime T cells and DCs for antitumor function [28]. T cells retained a degree of memory in subsequent drug administrations [24], proving that this treatment approach deserves further exploration in larger studies.

Despite the promising effects of ICIs at the molecular level, most studies report a significant clinical impact in a small fraction of patients, that have experienced, in general, prolonged survival and disease stabilization [19,25]. The value of PD-L1 positivity in GBM is controversial and it does not necessarily appear to predict treatment response. First, no consensus analysis nor clinical study validated a PD-L1 threshold correlating it with therapeutic efficacy [30]. Moreover, Ndoum et al. analyzed the PD-L1 expression in samples from 94 GBM patients with both immunohistochemistry and flow cytometry. They found that although most tumors were positive for PD-L1 by testing with both techniques (61% with a minimum of 1% cells expressing PD-L1, 38% with a minimum of 5%), the amount of PD-L1-positive GBM cells in each tumor was relatively low and exceptionally heterogeneous among the different samples (median: 2.77%, range: 0–86%) [30]. In the KEYNOTE-028 study, Reardon et al. report PD-L1 expression (1% or more of cells) in approximately 60% of the tumors screened, but cannot establish a relationship between PD-L1 expression and therapeutic benefit after ICI treatment [13]. Taken together, these findings make the broad use of ICI monotherapy unlikely to benefit most GBM patients.

The immunosuppressive nature of the TME is also challenging to counteract with ICI monotherapy [31], as this approach restricts the target population mostly to T cells. Moreover, ICI monotherapy fails to induce a polyclonal immune response [24]. The only published study with dual checkpoint inhibition in GBM [22] did not perform T cell subset analyses to address this issue. Other cellular players in the TME deserve future research and therapeutic efforts. For instance, tumor-associated macrophages (TAMs), including microglia and monocyte-derived macrophages (MDMs), can form up to 50% of the TME [32,33,34,35,36]. GBM cells actively shape the nature of TAMs in several ways to enhance immunosuppression, tumor invasion, and proliferation [37]. Recent studies have identified several therapeutic targets for myeloid cells, including IDO1, CSF1-CSF1R, CD39-CD73, SIRPa-CD47, and AXL kinase, for which further clinical evaluation is awaited [37]. Future studies will rely on better biomarkers of response to ICI immunotherapy. In addition, these studies should explore the combinations of ICIs with other forms of immunotherapy or targeted therapy aimed at other cells of the TME to synergize their antitumor effects.

### 2.2. Isocitrate Dehydrogenase (IDH) Mutant Gliomas

Isocitrate dehydrogenase (IDH)-mutant glioma is the second most common type of adult malignant glioma [38], and the most common type diagnosed in individuals younger than 50. IDH, a metabolic enzyme that catalyzes the conversion of isocitrate into α-ketoglutarate, when mutated, produces the oncometabolite D-2-hydroxyglutarate (D-2-HG) [38]. D-2-HG acts as an inhibitor of tumor suppressors and promotes epigenetic changes that lead to oncogene overexpression.

In addition, D-2-HG induces profound changes in T cell activity, proliferation, and metabolism [39]. As IDH-mutant gliomas produce D-2-HG in high amounts (in the mM range), this molecule then diffuses in paracrine fashion to tumor-infiltrating T cells, which uptake it regardless of their activation status through a solute carrier (SLC) transporter. Once inside the T cell, D-2-HG blocks the early phases of TCR signaling by downregulating the PLC-γ1-PIP2-NFAT-NFkB pathway. The reduction in NFAT nuclear translocation results in lower cytokine production (especially IFN-γ) and PD-1 expression [39]. Other studies have indicated a decrease in PD-L1 expression [40] and D-2-HG-related epigenetic suppression of PD-1 and PD-L1 by DNA methylation in IDH-mutant gliomas [41,42]. These findings may help explain the immunosuppressive TME in IDH-mutant gliomas and motivate previously reported instances of ICI treatment failure [39].

At the metabolic level, D-2-HG impairs polyamine biosynthesis by inhibiting ATP synthase ATP5B and altering its downstream signaling (AMPK-ODC1) [39]. The net effect is an impairment in T cell proliferation. Moreover, D-2-HG changes the profile of tumor-infiltrating immune cells, reducing CD8+ and memory T cells while increasing the proportion of naive CD4 + T cells [39]. These results indicate a selective impairment of the primary effector phase of T cell proliferation [39].

In conclusion, IDH-mutant gliomas are tumors with peculiar immunosuppressive features that may be more suitable to targeted therapy rather than immunotherapy. The results from several ongoing clinical trials (NCT03557359, NCT03893903, NCT04056910, and NCT02968940) using ICIs in IDH-mutant gliomas may either confirm or refute this hypothesis. In light of the immunosuppressive effect of D-2-HG, the idea of using ICIs concomitantly with IDH inhibitors (IDHi) has been put forth [39]. Still, the literature reports only a few accounts of this combination strategy, Table 2, and none had robust clinical benefits [43,44]. Therefore, this strategy warrants additional evaluation with larger clinical trials, some of which are already ongoing (NCT05484622).

**Table 2:** Selected studies on the use of ICI for the treatment of IDH-mutant gliomas.

Author	Phase	Regimen	Treatment
NCT03557359	II	Anti PD-1 monotherapy	Nivolumab
NCT03893903	I	IDH vax + anti-PD-L1	IDH-1 vaccine + Avelumab
NCT04056910	II	Anti-PD-1 + IDH1-inhibitor	Nivolumab + Ivosidenib
NCT02968940	II	Anti-PDL1 + radiation	Avelumab + HFRT

HFRT: hypo-fractionated radiotherapy; IDH1: isocitrate dehydrogenase 1.

**Table 3:** Selected studies on the use of ICI for the treatment of meningioma.

Author	Phase	Patients	Treatment
Bi et al., 2022 [45]	II	25 patients with recurrent grade 2 or 3 meningioma	Nivolumab
Brastianos et al., 2022 [46]	II	25 patients with grade 2 or 3 meningioma.	Pembrolizumab
Nidamanuri and Drappatz, 2022 [47]	Retrospective	8 patients with meningiomas	Anti-PD1 therapy

### 2.3. Meningioma

Although the vast majority of meningiomas are grade 1 and essentially cured after gross-total resection, grade 2 and, particularly grade 3, meningiomas experience regrowth after resection and radiation therapy, making them difficult to treat and leading to significant morbidity. Alkylating chemotherapy and targeted therapy are not generally considered effective for the management of high-grade meningiomas [48]. The rationale for ICI use in meningioma (Table 3) is based on the expression of immune checkpoint molecules, including CTLA-4, PD-1, and PD-L1 in these tumors, with PD-L1 expression being proportional to tumor grade [49,50,51].

In the first study using PD-1 blockers in meningioma treatment [45], 25 patients with recurrent grade 2 or 3 meningioma received nivolumab monotherapy in an open-label phase II trial. The treatment was well tolerated overall with a similar adverse effect profile as in other ICI monotherapy studies. Although the study did not meet its primary endpoint of progression-free survival at 6 months (PFS-6), the median PFS of 5.56 months was comparably higher than the median PFS in prior studies evaluating other therapies for high-grade meningiomas [48]. Supratentorial location and smaller tumor volume significantly correlated with improved OS. Conversely, skull-base location correlated with poorer outcomes, in line with previous studies highlighting the challenge of achieving gross total resection for tumors in this location [52,53]. At the cellular level, nivolumab did not induce substantial changes in TME composition and immune cell infiltration in samples from 13 patients with low tumor mutation burden (TMB < 10/Mb). Nonetheless, 2 patients with heavily pre-treated grade 3 meningioma displayed high TMB and experienced improved survival after treatment with nivolumab. Additionally, their TME characterization pre and post nivolumab treatment showed an increase in both CD4+ and CD8+ T cell counts. These findings indicate a selective therapeutic benefit of ICIs in those patients with high-grade meningiomas and a high TMB. This is consistent with previous studies highlighting the responsiveness of hypermutant tumors to immunotherapy [54].

In another prospective phase II trial, pembrolizumab showed promising results in a cohort of 25 patients with grade 2 or 3 meningiomas. The study achieved the primary endpoint with a PFS-6 rate of 48%. Moreover, the median PFS and OS were 7.6 months and 20.2 months, respectively. Notably, six patients had stabilization of meningioma growth and two patients had minor tumor regression which did not meet the criteria for partial response. Prior systemic therapy did not sensitize meningiomas to ICIs [46]. Interestingly, despite previous evidence of increased expression of PD-L1 and an immunosuppressive TME in high-grade meningiomas [51,55], this study found no significant correlation between PD-L1 expression in pre- and post-treated the meningioma samples and either PFS-6 rate or tumor growth curve stabilization, likely suggesting that other factors come into play at the level of TME [46]. Future studies should help elucidate the role of other cellular components of the TME on response. As noted above in the GBM section, one relevant example of these new targets is myeloid cells, as they have been reported to exert an immunosuppressive effect in the TME, thereby aiding tumor evasion of the immune system and likely hindering an efficient response to ICIs [56]. 

Nidamanuri and Drappatz conducted a retrospective study on eight meningioma patients undergoing ICI treatment for recurrent meningioma [47]. In this study, patients experienced a median PFS and median OS of 7 months and 1.75 years, respectively. These numbers increased in the subgroup of patients with grade 3 meningioma, up to 15 months and 2.5 years median PFS and mOS, respectively. Finally, responses were further improved in those patients with positive expression of PD-L1. This last finding contrasts with the results from Brastianos et al. and should be interpreted with caution given the small sample size and the selection and sampling bias, with this study being retrospective.

Overall, there appears to be an emerging role for ICI therapy in grade 2 and 3 meningiomas refractory to surgical and radiation treatment. The analysis of biomarkers such as PD-L1 expression, TMB, and immune cell profiling on patient samples should be considered to identify patients that are most likely to benefit from upfront, adjuvant treatment with ICIs.

### 2.4. Primary Central Nervous System Lymphoma

Primary central nervous system lymphoma (PCNSL) is a rare and aggressive form of extra-nodal non-Hodgkin lymphoma (NHL) originating in the brain, spinal cord, eye, or leptomeninges without systemic involvement [57,58]. This tumor occurs preferentially in older patients, with a median age at diagnosis of 67 years [59]. Immunosuppression is a risk factor for disease development, primarily due to Epstein–Barr virus (EBV) reactivation [58,60]. Although usually responsive to first-line chemotherapy with high-dose methotrexate, PCNSL has a 15% chance of treatment refractoriness [61] and a 36–66% relapse rate [62]. Overall, this tumor has a poor prognosis, with a median survival of 2 months, 7.2 months, and 2 years in untreated, relapsed, and all-cause scenarios, respectively [62].

Several observations underlie the rationale behind the use of ICIs in PCNSL. First, EBV induces PD-L1 overexpression in EBV-associated lymphomas [63]. Moreover, copy number gains and chromosomal translocations at chromosome 9p24.1, where the PD-L1/PD-L2 locus lies [64,65], are frequent in PCNSL [65]. Finally, a high TMB, caused by aberrant somatic hypermutation (aSHM) in PCNSL cells, is correlated with increased expression of PD-L1 [66]. Monabati et al. performed a retrospective analysis of PCNSL samples, reporting high PD-1 expression in TILs, as well as high PD-L1 expression in tumor cells [67]. Other authors have reported instances of PD-1/PD-L1 positivity in PCNSL samples on both tumor cells or TME cells, although the percentages of positive cells varied across the studies [66,68,69,70,71,72]. Overall, these findings support a potential therapeutic role of ICI in PCNSL, forming the basis for the ongoing clinical trials.

Four studies evaluated PD-1 inhibitors, including camrelizumab, sintilimab, and tislelizumab, alone or combined with other chemo-immunotherapy agents (NCT04688151, NCT04899427, NCT04052659, and NCT04070040). Moreover, one study employed dendritic cell (DC) vaccination in combination with nivolumab [69]. Most data available at this time for the use of ICIs in PCNSL come from case reports and small retrospective case series (see Table 4). However, among these are encouraging results, such as a case report demonstrating complete remission and clinical amelioration in a patient with poor performance status [73]. A more informed perspective on ICI use in this type of tumor will emerge from the ongoing studies listed in Table 4. Moving forward, it will be critical to establish the efficacy of ICI both as an induction treatment and as part of maintenance and consolidation regimens.

## 3. ICI for Brain Metastases

### 3.1. Parenchymal Metastases

Metastases are the most common brain tumors in adults, accounting for approximately 50% of brain tumor cases. Brain metastases (BM) most commonly arise from melanoma, breast cancer, and lung cancer [77] and historically had a dismal prognosis, with a median overall survival of approximately six months [78]. BM biology is peculiar, with significant differences in genetic and epigenetic alterations compared to their tumors of origin and even from other sites of dissemination [79,80]. The TME of BM also features unique T cell subclones compared to the primary tumors [81] and more immunosuppressive features compared to extracranial metastases [82,83], indicating that the cellular composition of the BM TME may be different or adapted explicitly for brain tissue. These findings shed some light on the clinically heterogeneous therapeutic response of BM [84], including for the treatment with ICIs.

### 3.2. Melanoma

Prior to the introduction of ICIs, the median survival of patients with melanoma brain metastases (MBM) treated with surgery, radiation and systemic therapies was in the order of four to five months [85]. The first trial with ICIs in MBM utilized ipilimumab in monotherapy in two arms, asymptomatic and symptomatic patients or patients under steroid therapy. This study provided initial signals of safety and efficacy of ICI therapy in melanoma BM [86]. Two landmark studies [87,88] investigated the combination of ipilimumab and nivolumab in MBM. Both studies demonstrated safety and durable intracranial responses in asymptomatic MBM cases. In CheckMate204, asymptomatic patients experienced an intracranial clinical benefit rate (CBR) of 58.4% and an intracranial PFS at 6 months (PFS-6) rate of 6.26%, while symptomatic patients demonstrated an intracranial CBR of 22.2% and a PFS-6 rate of 18.9%. Instead, for the study by Long et al., the median OS for the arm of the ipilimumab–nivolumab combination was not reached [88].

Thanks to the introduction of this combination ICI regimen, the 1-year OS rate for patients with MBM has changed from 25% to 85% [85]. Nonetheless, patients with symptomatic MBM had a significantly poorer prognosis across all trials and all measured outcomes, Table 5 [85,89].

A number of combination immunotherapy and targeted or radiation therapy studies are ongoing. Targeted therapy is thought to reduce the immunosuppressive features of the TME [96], thereby potentiating the action of ICI. In terms of radiation therapy, stereotactic radiosurgery (SRS) is preferred to whole-brain radiation therapy (WBRT) for patients with a discrete number of brain lesions [97,98]. WBRT is favored in leptomeningeal disease cases, multiple metastatic lesions, or recurrence after SRS [85]. The timing of RT with respect to ICI administration also has a potential implication for clinical outcomes. Additionally, Pomeranz et al. demonstrated a better prognosis in those MBM patients undergoing RT before ICIs [99]. Future clinical efforts should focus on identifying the optimal sequence of therapeutic interventions (including ICI, RT, and targeted therapy) to maximize the efficacy and the improvement of survival in these patients.

### 3.3. Breast Cancer

Breast cancer brain metastases (BCBM) represent the second most common type of BM overall [100]. Among the different subtypes of breast cancer, those that are estrogen receptor (ER)-negative, progesterone receptor (PR)-negative, and human epidermal growth factor receptor 2 (HER2)-negative-triple-negative breast cancer (TNBC)—are the most likely to lead to CNS metastases [101]. Systemic chemotherapy is often of limited benefit in this case, given poor drug penetration into the CNS and the presence of resistant clones among metastases.

IMpassion130 evaluated patients with metastatic triple negative breast cancer who underwent randomization between atezolizumab + nab-paclitaxel vs. placebo + nab-paclitaxel [102]. The study demonstrated an acceptable level of safety and an AE profile consistent with previous findings concerning atezolizumab. More importantly, overall, it showed a statistically significant therapeutic benefit against the placebo/chemotherapy arm, with a PFS of 7.2 months vs. 5.5 months and an OS of 21 months vs. 18.7 months, rising to 7.5 vs. 5 months for PFS and 25.0 vs. 15.5 months in PD-L1-positive tumors. Unfortunately, no clear conclusions could be drawn regarding the effectiveness of the ICI + nab-paclitaxel combination in BCBM patients due to the small sample size (only 15 patients out of 902), as patients with active or untreated brain metastases were excluded. The more recent KEYNOTE-355 study in advanced triple-negative breast cancer, also demonstrated the benefit of adding pembrolizumab to chemotherapy, but again excluded patients with active or untreated brain metastases, preventing the assessment of effectiveness in the BCBM patient population [103]. Dedicated studies of ICI therapy in patients with BCBM are ongoing (NCT02886585, NCT03417544) and will hopefully shed light on the role of this therapy in this patient population.

### 3.4. Non-Small Cell Lung Cancer

Non-small cell lung cancer (NSCLC) is the most common primary tumor with CNS dissemination [104,105]. Approximately 20% of NSCLC patients present with BM at diagnosis, and 25–50% develop BM throughout the disease [77,106,107]. If untreated, overall survival is just 1–2 months [108]. Moreover, the risk of brain dissemination is higher in those tumors driven by oncogenes, including *EGFR*, *ALK*, *ROS1*, and *KRAS* [109,110]. When it is possible to target these mutations, patients demonstrate good responses to targeted therapy with tyrosine kinase inhibitors (TKIs) [111,112,113,114,115,116,117]. However, a significant fraction of NSCLC is not driven by a specific oncogene [84] and therefore cannot benefit from targeted therapy.

In non-oncogene-driven NSCLC, ICIs play a central role and are currently the first-line treatment [84,118]. Overall, ICIs have shown promising results in NSCLC, with trials demonstrating a median OS of close to 4 years in select patient groups, Table 6 [84]. Those NSCLC cases with a PD-L1 expression of a minimum of 50% seem to benefit the most from ICI treatment [119]. Nonetheless, limitations in most of the clinical studies performed so far constrain our understanding of ICI efficacy in NSCLC. Specific limitations include: (i) patients with BM were systematically excluded from most studies [84,120] or analyzed only in subgroup analyses for which they represented less than 20% of the samples [121,122,123,124], (ii) the analyzed BM samples are small and suffer selection biases and poorly-defined inclusion criteria [125], (iii) the intracranial efficacy of ICI has so far been assessed with retrospective studies only, (iv) no studies have addressed the efficacy of ICI in patients with symptomatic BM, and (v) there is limited data about ICI monotherapy and CNS efficacy [126]. CheckMate 227, evaluating the combination of ipilimumab and nivolumab in patients with advanced NSCLC, did enroll a large number of patients with brain metastases (81/793) and did show a trend towards benefits in this subgroup [127]. Therefore, despite the large amount of clinical data on ICI use in NSCLC and emerging evidence of effectiveness in patients with BMs, dedicated randomized studies in BM patients are still lacking to fully characterize their effectiveness and identify those most likely to benefit from ICI therapy.

### 3.5. Leptomeningeal Metastases

Leptomeningeal metastases (LM) are a grave complication of metastatic disease, characterized by the dissemination of tumor cells to the leptomeninges and the cerebrospinal fluid (CSF). The prognosis is dismal, with an average survival of 3–7 weeks [131]. Approximately 10% of solid tumors and 5–15% of hematologic cancer can lead to LM, and the in increasing prevalence of LM is thought to be due to improved therapeutic success in managing systemic malignancies [132,133,134,135]. Lung, breast, and melanoma are the most common causes of LM. Moreover, LM can occur alone or concomitantly with parenchymal BM [131]. The presentation is widely heterogeneous, including headache, nausea and vomiting, gait instability, raised intracranial pressure, focal deficits (such as cranial nerve deficits, cauda equina syndrome, and radiculopathies), and more generalized symptoms like seizures and encephalopathy [132]. Current therapies have so far failed to produce substantial improvements in survival, are not standardized, and are mainly aimed at symptom palliation [131,132]. Radiation therapy is palliative and systemic and intrathecal therapy have produced mixed results, likely due to the heterogeneity of LM biology and selection bias in clinical trials [132]. In general poor patient performance status at the time of diagnosis, and the typical onset of resistance due to the high amount of previous treatment further complicate setting up dedicated clinical protocols for LM, leading to the exclusion of LM patients from most studies [131].

Brastianos and colleagues have suggested that the response rate of parenchymal BM to ICIs mirrors the ability of the immune system to overcome the anatomic barriers of the CNS [132], thereby holding promise for the treatment of LM as well. In this context, two histology-agnostic landmark trials (Table 7) have reported encouraging results of ICI therapy in LM. The first study was a phase II trial investigating the efficiency of ipilimumab-nivolumab combination in 18 patients suffering from LM [131]. This trial met its primary endpoint, with 44% overall survival at three months (OS3). The treatment was well tolerated, with only two patients discontinuing the protocol due to unacceptable toxicity (one for hepatitis and one for colitis).

The median survival was 2.9 months, the median intracranial PFS was 1.93 months, and the cumulative intracranial incidence-to-time progression at three months was 45%. Notably, the authors considered the role of concomitant corticosteroid therapy in 78% of enrolled patients as both mitigating the strength of some AEs and diminishing the efficacy of ICI. This study’s limitations included its small sample size, variable ICI dosing and administration schedule (according to tumor histology), and a relatively low representation of histologies such as melanoma and NSCLC.

In a second histology-agnostic phase II trial, 20 heavily pre-treated patients with LM received pembrolizumab monotherapy once every three weeks [132]. This study also met its primary endpoint, with a 60% OS3. Additionally, the median survival was 3.6 months, lowering to 2.4 months in patients receiving dexamethasone at enrolment. Interestingly, for BC-related LM, the outcome was not influenced by receptor status (ER/PR/HER2). The generalizability of the results to most LM histologies is limited given that 85% of enrolled patients had BC.

## 4. Discussion

The introduction of immune checkpoint inhibitors has ushered a new era for the treatment of systemic malignancies. This form of immunotherapy is also finding its way into treatment protocols for the management of brain tumors, with examples of marked success for the treatment of parenchymal brain metastases (particularly melanoma brain metastases) and initial encouraging results for high-grade meningioma, primary CNS lymphoma, and leptomeningeal metastases. For glioblastoma and IDH-mutant gliomas, no definitive clinical benefit has yet been demonstrated.

It is important to note that the quality of evidence supporting the use of ICIs for the treatment of brain tumors varies by tumor type, as the ICI treatment response of many brain tumors (including IDH-mut gliomas and several BM histologies) has yet to be reported with prospective, randomized clinical trials. For some of the tumors we reviewed, the evidence is limited to small retrospective case series, whose results need to be interpreted with caution. For these, additional prospective studies are warranted to validate the observed results.

In addition to clinical evaluation of the effectiveness of the ICI therapy, multiple questions remain as to the identification of biomarkers (such as tumor molecular alterations and characterization of the TME) enabling the selection of responders to therapy in light of the limited utility of PD-L1 expression, as well as the timing of ICI therapy with respect to other interventions (e.g., prior to surgical resection; concurrent or after radiation therapy), and its addition to other therapies, such as alkylating chemotherapy and targeted therapies. Lastly, the improved effectiveness of ICI therapy in glioma and other brain tumors will also depend on additional approaches for modulating immunosuppression in the tumor microenvironment (e.g., macrophage reprogramming) as well as steroid-sparing therapies for the management of peritumoral inflammation, since steroid use has been shown to blunt the response to ICI [136].

## Figures and Tables

**Table 1:** Selected studies on the use of ICI for the treatment of glioblastoma.

Author	Phase	Patients	Trial Arms	Outcomes
KeyNote-028 [13]	Ib	26 patients with PD-L1-positive recurrent GBM	Pembrolizumab	Investigation-assessed ORR (by RECIST v1.1): 8%. DOR: 8.3 and 22.8 months in 2 patients, respectively; mPFS: 2.8 months; PFS6 rate: 37.7% mOS: 13.1 months; OS rate at 12 months: 58%
Nayak L et al., 2021 [17]	II	80 patients with recurrent GBM	A: Pembrolizumab + Bevacizumab B: Pembrolizumab	A: 20% ORR, 26% PFS-6 rate;mOS: 8.8 months; B: 0% ORR
Nayak et al., 2022 [18]	II	137 patients with newly diagnosed and recurrent GBM	A: Durvalumab + RT B: Durvalumab B2: Durvalumab + Bevacizumab B3 and C: Durvalumab + low-dose Bevacizumab	Primary endpoints: A: OS-12 (not met) B, B2, B3: PFS-6 (not met) C: OS-6 (not met)
Lukas R et al., 2018 [19]	Ia	16 patients with recurrent GBM	Atezolizumab	Treatment safe and tolerated. 6% ORR; mPFS: 1.2 months; mOS: 4.2 months;
Chiocca EA et al. 2021 [20]	I	21 patients with recurrent GBM	A: Nivolumab 1 mg/kg + 10 mg VDX; B: Nivolumab 3 mg/kg + 10 mg VDX C: Nivolumab 3 mg/kg + 20 mg VDX	Treatment safe and tolerated. A and B: mOS: 16.9 months; C: mOS: 8.5 months
Omuro et al., 2022 (CheckMate-143) [21]	I	136 patients with newly diagnosed GBM	A: Nivolumab + TMZ + RT B: Nivolumab + RT	Primary endpoints: safety and tolerability (met; more frequent lymphopenia in cohort A); 2ry endpoint: OS (similar between A and B, different according to MGMT methylation status); Exploratory endpoints: PFS
CheckMate 498 [22]	III	560 patients with newly diagnosed MGMTunm GBM	A. Nivolumab + RT B: TMZ + RT	Treatment safe and tolerated. A: mOS: 13.4 months; PFS: 6 months; grade 3/4 AEs: 21.9%; serious AEs: 17.3%; B: mOS: 14.9 months; PFS: 6.2 months; grade 3/4 AEs; 25.1%; serious AEs: 7.6%
Schalper K et al., 2019 [23]	II	30 patients with resectable GBM (twenty-seven recurrent and three newly diagnosed)	Neoadjuvant Nivolumab	Safe and tolerable treatment. mPFS: 4.1 months; mOS: 7.3 months
Cloughesy T et al., 2019 [24]	NA	32 patients with recurrent GBM (53% MGMTm, 34% MGMTunm, and 13% unknown)	A: Neoadjuvant + adjuvant Pembrolizumab B. adjuvant Pembrolizumab	Treatment safe and tolerated. mOS: 13.7 vs. 7.5 months (A vs. B); mPFS: 3.3 vs. 2.4 months (A vs. B)
Reardon et al., 2020 [25]	III	369 with recurrent GBM (23.4% MGMTm, 22.7% MGMTunm, and 36.2% unknown)	A: Nivolumab B: Bevacizumab	Primary endpoint not met. Nivolumab safe and tolerated. A: mOS: 9.8 months; ORR: 7.8%; B: mOS: 10 months; ORR: 23.1%; 1-yr OS: 42% for both groups;grade 3/4 AEs similar in A and B
CheckMate 548 [26]	III	716 patients with newly diagnosed MGMTm GBM	A: Nivolumab + TMZ + RT B: PBO + TMZ +RT	Treatment safe and tolerated. mPFS: 10.6 vs. 10.3 months (A vs. B); mOS: 28.9 vs. 32.1 months (A vs. B); with basal corticosteroids, mOS: 31.3 vs. 33 months (A vs. B)
Checkate-143 [22]	I	40 patients with recurrent GBM	A: Nivolumab 3mg/kg B. Ipilimumab 3 mg/kg + Nivolumab 1 mg/kg C: Ipilimumab 1mg/kg+ Nivolumab 3 mg/kg	Nivolumab monotherapy better tolerated than Ipilimumab-nivolumab combo; mPFS: 1.9 vs. 1.5 vs. 2.1 months (A vs. B vs. C); mOS: 10.4 vs. 9.2 vs. 7.3 months (A vs. B vs. C)

GBM: glioblastoma; TME: tumor microenvironment; PBO: placebo; TMZ: temozolomide; RT: radiotherapy; VDX: Veledimex; RECIST v1.1: Response Evaluation Criteria In Solid Tumors, version 1.1; AE: adverse events; OS: overall survival; mOS: median overall survival; PFS: progression-free survival; mPFS: median progression-free survival; DOR: duration of response; MGMT: O6-methylguanine-DNA-methyltransferase; MGMTm: methylated O6-methylguanine-DNA-methyltransferase; MGMTunm: unmethylated O6-methylguanine-DNA-methyltransferase; ORR: objective response rate; PFS-6: progression-free survival at 6 months; OS-12: overall survival at 12 months.

**Table 4:** Selected studies on the use of ICI for the treatment of primary CNS lymphoma.

Author or Trial Name	Phase	Drug	Cohort	Results
Furuse et al., 2017 [69]	Case report	Nivolumab + DC vaccination	1 patient	CR maintained for 10 months
Nayak et al., 2017 [57]	Case series	Nivolumab	5 patietns (4 with PCNSL and 1 with PTL)	4 patients with CR and 1 with PR;
Graber J et al., 2020 [74]	Case series	Pembrolizumab	5 patients (PCNSL and SCNSL)	Prolonged remission in 3 out of 5 patients
Ambady et al., 2019 [75]	Retrospective study	Nivolumab/Pembrolizumab and Rituximab	6 patients (three with PCNSL and three SCNSL)	3 out of 6 patients with CR
Gavrilenko A et al., 2020 [75,76];	Case series	Nivolumab	8 patients with PCNSL and one with PTL	2-year OS: 44%; mOS: 12 months; 2-year PFS: 26%; mPFS: 12 months;

PCNSL: primary central nervous system lymphoma; SCNSL: secondary central nervous system lymphoma; PTL: primary testicular lymphoma; DC: dendritic cell; CR: complete response; PR: partial response; OS: overall survival; mOS: median overall survival; PFS: progression-free survival; mPFS: median progression-free survival; DOR: duration of response.

**Table 5:** Selected studies on the use of ICI for the treatment of melanoma brain metastases.

Study/Studies	Phase	Therapies	Patient Cohort(s)	Results (ORR, PFS, OS)
Margolin et al., 2012 [86]	II	Ipilimumab	Cohort A: asymptomatic MBM (51); Cohort B: symptomatic MBM on (21)	iDCR: 24 vs. 10% (A vs. B); mPFS: 1.5–1.9 vs. 1.2 months (A vs. B); mOS: 7 vs. 3.7 months (A vs. B)
NIBIT M1 [90];	II	Ipilimumab + Fotemustine	20 patients with asymptomatic brain metastases out of a cohort of eight-six patients with advanced melanoma	In MBM patients: Brain-PFS: 3 months
NIBIT-M2 [91]	III	Ipilimumab, Nivolumab, Fotemustine	80 patients with MBM Arm A: Fotemustine (27) Arm B: Ipilimumab + Fotemustine (26) Arm C: Ipilimumab + Nivolumab (27)	mOS: 8.5 vs. 8.2 vs. 29.2 months (A vs. B vs. C); mPFS: 3 vs. 3 vs. 8.7 months (A vs. B vs. C); ICR: 0 vs. 19.2 vs. 44.4% (A vs. B vs. C)
Goldberg et al., 2016 [92]	II	Pembrolizumab	18 patients with MBM in a cohort of 52 patients with brain metastases	ORR: 22% mOS: not reached
Kluger et al., 2018; [93]	II	Pembrolizumab	23 patients with MBM	RR: 26% mPFS: 2 months; mOS: 17 months
Ascierto et al., 2017,2020 [94,95]	III	Ipilimumab A: 10 mg/kg; B: 3 mg/kg;	127 patients with MBMs in a cohort of 727 patients with advanced melanoma	In MBM patients: mOS: 7 months vs. 5.7 months (A vs. B)
Checkmate204 [89]	II	Nivolumab + Ipilimumab	119 patients with MBM Cohort A: asymptomatic (101); Cohort B: symptomatic (18)	iORR: 53.5% vs. 16.7% (A vs. B); 36-month iPFS: 54.1% vs. 18.9% (A vs. B); 36-month OS: 71.9% vs. 36.6% (A vs. B)
ABC study [88]	II	Nivolumab + Ipilimumab vs. Nivolumab	79 patients with MBM; Cohort A: Nivolumab + Ipilimumab (36) Cohort B: Nivolumab (27); Cohort C: Nivolumab prior Tx, symptomatic, or with LM (16);	ICR 46% vs. 20% vs. 6% (A vs. B vs. C); ICCR 17% vs. 12% vs. 0% (A vs. B vs. C); iPFS: not reached vs. 2.5 vs. 2.3 months (A vs. B vs. C); OS: not reached vs. 18.5 vs. 5.1 months (A vs. B vs. C)

MBM: melanoma brain metastases; LM: leptomeningeal disease; ORR: overall response rate; iORR: intracranial objective response rate; PFS: progression-free survival; mPFS: median progression-free survival; iPFS: intracranial progression-free survival; OS: overall survival; mOS: median overall survival; iDCR: intracranial disease control rate; ICR: intracranial response; ICCR: intracranial complete response; PD: progressive disease; CR: complete response rate; PR: partial response rate; RR: response rate; LMD: leptomeningeal disease.

**Table 6:** Selected studies on the use of ICI for the treatment of non-small cell lung cancer metastases.

Study	Phase	Therapy	Patient Cohort	Patients with BM	Results
Goldberg et al., 2020 [126]	II	Pembrolizumab	42 asymptomatic patients with untreated BM from NSCLC; Cohort A: PD-L1 expression ≥1%; Cohort B: PD-L1 expression <1% or unevaluable;	100%	Cohort A: 29.7% BM response rate; Cohort 2: no response
Keynote-189 study [128,129]	III	ICI Arm (A): Pembrolizumab + Pemetrexed + a Pt-based CT; Control Arm (B): Pemetrexed + a Pt-based CT;	108 patents among a cohort of six hundred and sixteen patients with metastatic n-sq-NSCLC	17.53%	For BM patients: mOS: 19.2 vs. 7.5 months (A vs. B); HR for OS (A vs. B): 0.41
Crinò et al., 2019 [125]	EAP	Nivolumab	409 patients with asymptomatic or controlled BM in a cohort of 1588 patients with advanced n-sq-NSCLC	26%	BM patients: mOS: 8.6 months; mPFS: 3 months; iDCR: 40%; ORR:17%
OAK trial [130]	III	ICI Arm (A): Atezolizumab; Control Arm (B): docetaxel	85 patients with BM in a cohort of 850 patients with previously treated stage IIIB/IV NSCLC;	10%	For BM patients: mOS: 20.1 vs. 11.9 months (A vs. B); OS HR for Atezolizumab: 0.54

NSCLC: non-small cell lung cancer; BM: brain metastases; EAP: expanded access program; Pt: platinum; CT: chemotherapy; n-sq: non-squamous; mOS: median overall survival; OS: overall survival mPFS: median progression-free survival; PFS: progression-free survival; HR: hazard ratio; ORR: objective response rate; iDCR: intracranial disease control rate.

**Table 7:** Selected studies on the use of ICI for the treatment of leptomeningeal metastases.

Author	Phase	Patients	Treatment
Brastianos et al., 2021 [131]	II	18 patients with LM	Ipilimumab–Nivolumab
Brastianos et al., 2020 [132]	II	20 patients with pretreated LM	Pembrolizumab monotherapy

LM: leptomeningeal metastases.

## Data Availability

No new data were created or analyzed in this study. Data sharing is not applicable to this article.

## References

[1] Wei S.C., Duffy C.R., Allison J.P. (2018). Fundamental Mechanisms of Immune Checkpoint Blockade Therapy. Cancer Discov..

[2] Twomey J.D., Zhang B. (2021). Cancer Immunotherapy Update: FDA-Approved Checkpoint Inhibitors and Companion Diagnostics. AAPS J..

[3] Rossi E., Schinzari G., Maiorano B.A., Indellicati G., Di Stefani A., Pagliara M.M., Fragomeni S.M., De Luca E.V., Sammarco M.G., Garganese G. (2021). Efficacy of immune checkpoint inhibitors in different types of melanoma. Hum. Vaccin. Immunother..

[4] Berghmans T., Durieux V., Hendriks L.E.L., Dingemans A.M. (2020). Immunotherapy: From Advanced NSCLC to Early Stages, an Evolving Concept. Front. Med..

[5] Tomaszewski W., Sanchez-Perez L., Gajewski T.F., Sampson J.H. (2019). Brain Tumor Microenvironment and Host State: Implications for Immunotherapy. Clin. Cancer Res..

[6] Medikonda R., Dunn G., Rahman M., Fecci P., Lim M. (2021). A review of glioblastoma immunotherapy. J. Neuro-Oncol..

[7] Sampson J.H., Gunn M.D., Fecci P.E., Ashley D.M. (2020). Brain immunology and immunotherapy in brain tumours. Nat. Rev. Cancer.

[8] Stupp R., Mason W.P., van den Bent M.J., Weller M., Fisher B., Taphoorn M.J.B., Belanger K., Brandes A.A., Marosi C., Bogdahn U. (2005). Radiotherapy plus Concomitant and Adjuvant Temozolomide for Glioblastoma. N. Engl. J. Med..

[9] Stupp R., Hegi M.E., Mason W.P., Van Den Bent M.J., Taphoorn M.J., Janzer R.C., Ludwin S.K., Allgeier A., Fisher B., Belanger K. (2009). Effects of radiotherapy with concomitant and adjuvant temozolomide versus radiotherapy alone on survival in glioblastoma in a randomised phase III study: 5-year analysis of the EORTC-NCIC trial. Lancet Oncol..

[10] Lamborn K.R., Yung W.K.A., Chang S.M., Wen P.Y., Cloughesy T.F., DeAngelis L., Robins H.I., Lieberman F.S., Fine H.A., Fink K.L. (2008). Progression-free survival: An important end point in evaluating therapy for recurrent high-grade gliomas. Neuro-Oncol..

[11] Wu W., Lamborn K.R., Buckner J.C., Novotny P.J., Chang S.M., O’Fallon J.R., Jaeckle K.A., Prados M.D. (2009). Joint NCCTG and NABTC prognostic factors analysis for high-grade recurrent glioma. Neuro-Oncol..

[12] Clarke J.L., Ennis M.M., Yung W.K.A., Chang S.M., Wen P.Y., Cloughesy T.F., DeAngelis L., Robins H.I., Lieberman F.S., Fine H.A. (2011). Is surgery at progression a prognostic marker for improved 6-month progression-free survival or overall survival for patients with recurrent glioblastoma?. Neuro-Oncol..

[13] Reardon D.A., Kim T.M., Frenel J., Simonelli M., Lopez J., Subramaniam D.S., Siu L.L., Wang H., Krishnan S., Stein K. (2021). Treatment with pembrolizumab in programmed death ligand 1–positive recurrent glioblastoma: Results from the multicohort phase 1 KEYNOTE-028 trial. Cancer.

[14] Berghoff A.S., Kiesel B., Widhalm G., Rajky O., Ricken G., Wöhrer A., Dieckmann K., Filipits M., Brandstetter A., Weller M. (2015). Programmed death ligand 1 expression and tumor-infiltrating lymphocytes in glioblastoma. Neuro-Oncol..

[15] Wilmotte R., Burkhardt K., Kindler V., Belkouch M.-C., Dussex G., De Tribolet N., Walker P.R., Dietrich P.-Y. (2005). B7-homolog 1 expression by human glioma: A new mechanism of immune evasion. Neuroreport.

[16] Xue S., Song G., Yu J. (2017). The prognostic significance of PD-L1 expression in patients with glioma: A meta-analysis. Sci. Rep..

[17] Nayak L., Molinaro A.M., Peters K.B., Clarke J.L., Jordan J.T., de Groot J.F., Nghiemphu P.L., Kaley T.J., Colman H., McCluskey C. (2021). Randomized Phase II and Biomarker Study of Pembrolizumab plus Bevacizumab versus Pembrolizumab Alone for Patients with Recurrent Glioblastoma. Clin. Cancer Res..

[18] Nayak L., Standifer N., Dietrich J., Clarke J.L., Dunn G.P., Lim M., Cloughesy T., Gan H.K., Flagg E., George E. (2022). Circulating Immune Cell and Outcome Analysis from the Phase II Study of PD-L1 Blockade with Durvalumab for Newly Diagnosed and Recurrent Glioblastoma. Clin. Cancer Res..

[19] Lukas R.V., Rodon J., Becker K., Wong E.T., Shih K., Touat M., Fassò M., Osborne S., Molinero L., O’Hear C. (2018). Clinical activity and safety of atezolizumab in patients with recurrent glioblastoma. J. Neuro-Oncol..

[20] Chiocca E.A., Gelb A.B., Chen C.C., Rao G., A Reardon D., Wen P.Y., Bi W.L., Peruzzi P., Amidei C., Triggs D. (2021). Combined immunotherapy with controlled interleukin-12 gene therapy and immune checkpoint blockade in recurrent glioblastoma: An open-label, multi-institutional phase I trial. Neuro-Oncol..

[21] Omuro A., A Reardon D., Sampson J.H., Baehring J., Sahebjam S., Cloughesy T.F., Chalamandaris A.-G., Von Potter V., Butowski N., Lim M. (2022). Nivolumab plus radiotherapy with or without temozolomide in newly diagnosed glioblastoma: Results from exploratory phase I cohorts of CheckMate 143. Neuro-Oncol. Adv..

[22] Omuro A., Vlahovic G., Lim M., Sahebjam S., Baehring J., Cloughesy T., Voloschin A., Ramkissoon S.H., Ligon K.L., Latek R. (2018). Nivolumab with or without ipilimumab in patients with recurrent glioblastoma: Results from exploratory phase I cohorts of CheckMate 143. Neuro-Oncol..

[23] Schalper K.A., Rodriguez-Ruiz M.E., Diez-Valle R., López-Janeiro A., Porciuncula A., Idoate M.A., Inogés S., De Andrea C., De Cerio A.L.-D., Tejada S. (2019). Neoadjuvant nivolumab modifies the tumor immune microenvironment in resectable glioblastoma. Nat. Med..

[24] Cloughesy T.F., Mochizuki A.Y., Orpilla J.R., Hugo W., Lee A.H., Davidson T.B., Wang A.C., Ellingson B.M., Rytlewski J.A., Sanders C.M. (2019). Neoadjuvant anti-PD-1 immunotherapy promotes a survival benefit with intratumoral and systemic immune responses in recurrent glioblastoma. Nat. Med..

[25] Reardon D.A., Brandes A.A., Omuro A., Mulholland P., Lim M., Wick A., Baehring J., Ahluwalia M.S., Roth P., Bähr O. (2020). Effect of Nivolumab vs Bevacizumab in Patients With Recurrent Glioblastoma: The CheckMate 143 Phase 3 Randomized Clinical Trial Supplemental content. JAMA Oncol..

[26] Lim M., Weller M., Idbaih A., Steinbach J., Finocchiaro G., Raval R.R., Ansstas G., Baehring J., Taylor J.W., Honnorat J. (2022). Phase III trial of chemoradiotherapy with temozolomide plus nivolumab or placebo for newly diagnosed glioblastoma with methylated *MGMT* promoter. Neuro-Oncol..

[27] Arjaans M., Oosting S.F., Schröder C.P., de Vries E.G. (2013). Bevacizumab-Induced Vessel Normalization Hampers Tumor Uptake of Antibodies—Response. Cancer Res..

[28] Lee A.H., Sun L., Mochizuki A.Y., Reynoso J.G., Orpilla J., Chow F., Kienzler J.C., Everson R.G., Nathanson D.A., Bensinger S.J. (2021). Neoadjuvant PD-1 blockade induces T cell and cDC1 activation but fails to overcome the immunosuppressive tumor associated macrophages in recurrent glioblastoma. Nat. Commun..

[29] Omuro A., A Brandes A., Carpentier A.F., Idbaih A., A Reardon D., Cloughesy T., Sumrall A., Baehring J., Bent M.V.D., Bähr O. (2022). Radiotherapy combined with nivolumab or temozolomide for newly diagnosed glioblastoma with unmethylated *MGMT* promoter: An international randomized phase III trial. Neuro-Oncol..

[30] Nduom E.K., Wei J., Yaghi N.K., Huang N., Kong L.-Y., Gabrusiewicz K., Ling X., Zhou S., Ivan C., Chen J.Q. (2016). PD-L1 expression and prognostic impact in glioblastoma. Neuro-Oncol..

[31] Wang P., Fang X., Yin T., Tian H., Yu J., Teng F. (2021). Efficacy and Safety of Anti-PD-1 Plus Anlotinib in Patients With Advanced Non–Small-Cell Lung Cancer After Previous Systemic Treatment Failure—A Retrospective Study. Front. Oncol..

[32] Klemm F., Maas R.R., Bowman R.L., Kornete M., Soukup K., Nassiri S., Brouland J.-P., Iacobuzio-Donahue C.A., Brennan C., Tabar V. (2020). Interrogation of the Microenvironmental Landscape in Brain Tumors Reveals Disease-Specific Alterations of Immune Cells. Cell.

[33] Phillips J.P., Eremin O., Anderson J.R. (1982). Lymphoreticuar cells in human brain tumors and in the normal brain. Br. J. Cancer..

[34] Darmanis S., Sloan S.A., Croote D., Mignardi M., Chernikova S., Samghababi P., Zhang Y., Neff N., Kowarsky M., Caneda C. (2017). Single-Cell RNA-Seq Analysis of Infiltrating Neoplastic Cells at the Migrating Front of Human Glioblastoma. Cell Rep..

[35] Gieryng A., Pszczolkowska D., Walentynowicz K.A., Rajan W.D., Kaminska B. (2017). Immune microenvironment of gliomas. Lab. Investig..

[36] Venteicher A.S., Tirosh I., Hebert C., Yizhak K., Neftel C., Filbin M.G., Hovestadt V., Escalante L.E., Shaw M.L., Rodman C. (2017). Decoupling genetics, lineages, and microenvironment in IDH-mutant gliomas by single-cell RNA-seq. Science.

[37] Andersen B.M., Akl C.F., Wheeler M.A., Chiocca E.A., Reardon D.A., Quintana F.J. (2021). Glial and myeloid heterogeneity in the brain tumour microenvironment. Nat. Rev. Cancer.

[38] Miller J.J., Gonzalez Castro L.N., McBrayer S., Weller M., Cloughesy T., Portnow J., Andronesi O., Barnholtz-Sloan J.S., Baumert B.G., Berger M.S. (2022). Isocitrate dehydrogenase (IDH) mutant gliomas: A Society for Neuro-Oncology (SNO) consensus review on diagnosis, management, and future directions. Neuro-Oncol..

[39] Bunse L., Pusch S., Bunse T., Sahm F., Sanghvi K., Friedrich M., Alansary D., Sonner J.K., Green E., Deumelandt K. (2018). Suppression of antitumor T cell immunity by the oncometabolite (R)-2-hydroxyglutarate. Nat. Med..

[40] Wang Z., Zhang C., Liu X., Wang Z., Sun L., Li G., Liang J., Hu H., Liu Y., Zhang W. (2016). Molecular and clinical characterization of PD-L1 expression at transcriptional level via 976 samples of brain glioma. Oncoimmunology.

[41] Mu L., Long Y., Yang C., Jin L., Tao H., Ge H., Chang Y.E., Karachi A., Kubilis P.S., De Leon G. (2018). The IDH1 Mutation-Induced Oncometabolite, 2-Hydroxyglutarate, May Affect DNA Methylation and Expression of PD-L1 in Gliomas. Front. Mol. Neurosci..

[42] Röver L.K., Gevensleben H., Dietrich J., Bootz F., Landsberg J., Goltz D., Dietrich D. (2018). PD-1 (PDCD1) Promoter Methylation Is a Prognostic Factor in Patients With Diffuse Lower-Grade Gliomas Harboring Isocitrate Dehydrogenase (IDH) Mutations. Ebiomedicine.

[43] Mellinghoff I.K., Ellingson B.M., Touat M., Maher E., Macarena I., Holdhoff M., Cote G.M., Burris H., Janku F., Young R.J. (2020). Ivosidenib in Isocitrate Dehydrogenase 1-Mutated Advanced Glioma. J. Clin. Oncol..

[44] Natsume A., Wakabayashi T., Miyakita Y., Narita Y., Mineharu Y., Arakawa Y., Yamasaki F., Sugiyama K., Hata N., Muragaki Y. (2019). Phase I study of a brain penetrant mutant IDH1 inhibitor DS-1001b in patients with recurrent or progressive *IDH1* mutant gliomas. J. Clin. Oncol..

[45] Bi W.L., Nayak L., Meredith D.M., Driver J., Du Z., Hoffman S., Li Y., Lee E.Q., Beroukhim R., Rinne M. (2022). Activity of PD-1 blockade with nivolumab among patients with recurrent atypical/anaplastic meningioma: Phase II trial results. Neuro-Oncol..

[46] Brastianos P.K., Kim A.E., Giobbie-Hurder A., Lee E.Q., Wang N., Eichler A.F., Chukwueke U., Forst D.A., Arrillaga-Romany I.C., Dietrich J. (2022). Phase 2 study of pembrolizumab in patients with recurrent and residual high-grade meningiomas. Nat. Commun..

[47] Nidamanuri P., Drappatz J. (2022). Immune checkpoint inhibitor therapy for recurrent meningiomas: A retrospective chart review. J. Neuro-Oncol..

[48] Kaley T., Barani I., Chamberlain M., McDermott M., Panageas K., Raizer J., Rogers L., Schiff D., Vogelbaum M., Weber D. (2014). Historical benchmarks for medical therapy trials in surgery- and radiation-refractory meningioma: A RANO review. Neuro-Oncol..

[49] Bi W.L., Greenwald N.F., Abedalthagafi M., Wala J., Gibson W.J., Agarwalla P.K., Horowitz P., Schumacher S.E., Esaulova E., Mei Y. (2017). Genomic landscape of high-grade meningiomas. NPJ Genom. Med..

[50] Huang J., Campian J.L., Gao F., Johanns T.M., Desjardins A., Wang-Gillam A., Rubin J. (2019). A phase I/II study of nivolumab plus or minus ipilimumab in combination with multifraction stereotactic radiosurgery for recurrent high-grade radiation-relapsed meningioma. J. Clin. Oncol..

[51] Li Y.D., Veliceasa D., Lamano J.B., Lamano J.B., Kaur G., Biyashev D., Horbinski C.M., Kruser T.J., Bloch O. (2019). Systemic and local immunosuppression in patients with high-grade meningiomas. Cancer Immunol. Immunother..

[52] Savardekar A.R., Patra D.P., Bir S., Thakur J.D., Mohammed N., Bollam P., Georgescu M.-M., Nanda A. (2018). Differential Tumor Progression Patterns in Skull Base Versus Non–Skull Base Meningiomas: A Critical Analysis from a Long-Term Follow-Up Study and Review of Literature. World Neurosurg..

[53] Meling T.R., Da Broi M., Scheie D., Helseth E. (2019). Meningiomas: Skull base versus non-skull base. Neurosurg. Rev..

[54] Nebot-Bral L., Brandao D., Verlingue L., Rouleau E., Caron O., Despras E., El-Dakdouki Y., Champiat S., Aoufouchi S., Leary A. (2017). Hypermutated tumours in the era of immunotherapy: The paradigm of personalised medicine. Eur. J. Cancer.

[55] Du Z., Abedalthagafi M., Aizer A.A., McHenry A.R., Sun H.H., Bray M.-A., Viramontes O., Machaidze R., Brastianos P.K., Reardon D.A. (2014). Increased expression of the immune modulatory molecule PD-L1 (CD274) in anaplastic meningioma. Oncotarget.

[56] De Cicco P., Ercolano G., Ianaro A. (2020). The New Era of Cancer Immunotherapy: Targeting Myeloid-Derived Suppressor Cells to Overcome Immune Evasion. Front. Immunol..

[57] Nayak L., Iwamoto F.M., LaCasce A., Mukundan S., Roemer M.G.M., Chapuy B., Armand P., Rodig S.J., Shipp M.A. (2017). PD-1 blockade with nivolumab in relapsed/refractory primary central nervous system and testicular lymphoma. Blood.

[58] Schaff L.R., Grommes C. (2021). Update on Novel Therapeutics for Primary CNS Lymphoma. Cancers.

[59] Ostrom Q.T., Patil N., Cioffi G., Waite K., Kruchko C., Barnholtz-Sloan J.S. (2020). CBTRUS Statistical Report: Primary Brain and Other Central Nervous System Tumors Diagnosed in the United States in 2013–2017. Neuro-Oncol..

[60] Zhang N., Zuo Y., Jiang L., Peng Y., Huang X., Zuo L. (2022). Epstein-Barr Virus and Neurological Diseases. Front. Mol. Biosci..

[61] Jahnke K., Thiel E., Martus P., Herrlinger U., Weller M., Fischer L., Korfel A., on behalf of the German Primary Central Nervous System Lymphoma Study Group (G-PCNSL-SG) (2006). Relapse of primary central nervous system lymphoma: Clinical features, outcome and prognostic factors. J. Neuro-Oncol..

[62] Tao K., Wang X., Tian X. (2021). Relapsed Primary Central Nervous System Lymphoma: Current Advances. Front. Oncol..

[63] Lin N., Song Y., Zhu J. (2020). Immune checkpoint inhibitors in malignant lymphoma: Advances and perspectives. Chin. J. Cancer Res..

[64] Chapuy B., Roemer M.G.M., Stewart C., Tan Y., Abo R.P., Zhang L., Dunford A.J., Meredith D.M., Thorner A.R., Jordanova E.S. (2016). Targetable genetic features of primary testicular and primary central nervous system lymphomas. Blood.

[65] Grommes C., Nayak L., Tun H.W., Batchelor T.T. (2018). Introduction of novel agents in the treatment of primary CNS lymphoma. Neuro-Oncol..

[66] Ou A., Sumrall A., Phuphanich S., Spetzler D., Gatalica Z., Xiu J., Michelhaugh S., Brenner A., Pandey M., Kesari S. (2020). Primary CNS lymphoma commonly expresses immune response biomarkers. Neuro-Oncol. Adv..

[67] Monabati A., Nematollahi P., Dehghanian A., Safaei A., Sadeghipour A., Movahedinia S., Mokhtari M. (2020). Immune Checkpoint Molecules in Primary Diffuse Large B-Cell Lymphoma of the Central Nervous System. Basic Clin. Neurosci. J..

[68] Berghoff A.S., Ricken G., Widhalm G., Rajky O., Hainfellner J.A., Birner P., Raderer M., Preusser M. (2014). PD1 (CD279) and PD-L1 (CD274, B7H1) expression in primary central nervous system lymphomas (PCNSL). Clin. Neuropathol..

[69] Furuse M., Nonoguchi N., Omura N., Shirahata M., Iwasaki K., Inui T., Kuroiwa T., Kuwabara H., Miyatake S.-I. (2017). Immunotherapy of Nivolumab with Dendritic Cell Vaccination Is Effective against Intractable Recurrent Primary Central Nervous System Lymphoma: A Case Report. Neurol. Med.-Chir..

[70] Panjwani P.K., Charu V., DeLisser M., Molina-Kirsch H., Natkunam Y., Zhao S. (2018). Programmed death-1 ligands PD-L1 and PD-L2 show distinctive and restricted patterns of expression in lymphoma subtypes. Hum. Pathol..

[71] Miyasato Y., Takashima Y., Takeya H., Yano H., Hayano A., Nakagawa T., Makino K., Takeya M., Yamanaka R., Komohara Y. (2018). The expression of PD-1 ligands and IDO1 by macrophage/microglia in primary central nervous system lymphoma. J. Clin. Exp. Hematop..

[72] Alame M., Pirel M., Costes-Martineau V., Bauchet L., Fabbro M., Tourneret A., De Oliveira L., Durand L., Roger P., Gonzalez S. (2020). Characterisation of tumour microenvironment and immune checkpoints in primary central nervous system diffuse large B cell lymphomas. Virchows Arch..

[73] El-Tawab R., Hamada A., Elhagracy R., Pinto K., Alshemmari S. (2020). Promising effect of PDL1 inhibitors in the front-line management of primary aggressive central nervous system lymphoma: A case report. Hematol. Oncol. Stem Cell Ther..

[74] Graber J.J., Plato B., Mawad R., Moore D.J. (2020). Pembrolizumab immunotherapy for relapsed CNS Lymphoma. Leuk. Lymphoma.

[75] Ambady P., Szidonya L., Firkins J., James J., Johansson K., White T., Jezierski C., Doolittle N.D., Neuwelt E.A. (2019). Combination immunotherapy as a non-chemotherapy alternative for refractory or recurrent CNS lymphoma. Leuk. Lymphoma.

[76] Gavrilenko A.N., Volkov N.P., Shmidt D.I., Polushin A.Y., Kondakova E., Lepik K.V., Zalaylov Y.R., Popova M.O., Kulagin A.D., Afanasyev B.V. (2020). Nivolumab in Primary CNS Lymphoma and Primary Testicular Lymphoma with CNS Involvement: Single Center Experience. Blood.

[77] Barnholtz-Sloan J.S., Sloan A.E., Davis F.G., Vigneau F.D., Lai P., Sawaya R.E. (2004). Incidence Proportions of Brain Metastases in Patients Diagnosed (1973 to 2001) in the Metropolitan Detroit Cancer Surveillance System. J. Clin. Oncol..

[78] Stelzer K. (2013). Epidemiology and prognosis of brain metastases. Surg. Neurol. Int..

[79] Brastianos P.K., Carter S.L., Santagata S., Cahill D.P., Taylor-Weiner A., Jones R.T., Van Allen E.M., Lawrence M.S., Horowitz P.M., Cibulskis K. (2015). Genomic Characterization of Brain Metastases Reveals Branched Evolution and Potential Therapeutic Targets. Cancer Discov..

[80] Orozco J.I.J., Knijnenburg T.A., Manughian-Peter A.O., Salomon M.P., Barkhoudarian G., Jalas J.R., Wilmott J.S., Hothi P., Wang X., Takasumi Y. (2018). Epigenetic profiling for the molecular classification of metastatic brain tumors. Nat. Commun..

[81] Mansfield A.S., Ren H., Sutor S., Sarangi V., Nair A., Davila J., Elsbernd L.R., Udell J.B., Dronca R.S., Park S. (2018). Contraction of T cell richness in lung cancer brain metastases. Sci. Rep..

[82] Fischer G.M., Jalali A., Kircher D.A., Lee W.-C., McQuade J.L., Haydu L.E., Joon A.Y., Reuben A., de Macedo M.P., Carapeto F.C.L. (2019). Molecular Profiling Reveals Unique Immune and Metabolic Features of Melanoma Brain Metastases. Cancer Discov..

[83] Fukumura K., Malgulwar P.B., Fischer G.M., Hu X., Mao X., Song X., Hernandez S.D., Zhang X.H.-F., Zhang J., Parra E.R. (2021). Multi-omic molecular profiling reveals potentially targetable abnormalities shared across multiple histologies of brain metastasis. Acta Neuropathol..

[84] Alvarez-Breckenridge C., Remon J., Piña Y., Nieblas-Bedolla E., Forsyth P., Hendriks L., Brastianos P.K. (2022). Emerging Systemic Treatment Perspectives on Brain Metastases: Moving Toward a Better Outlook for Patients. Am. Soc. Clin. Oncol. Educ. Book.

[85] Tawbi H.A., Forsyth P.A., Algazi A., Hamid O., Hodi F.S., Moschos S.J., Khushalani N.I., Lewis K., Lao C.D., Postow M.A. (2018). Combined Nivolumab and Ipilimumab in Melanoma Metastatic to the Brain. N. Engl. J. Med..

[86] Margolin K., Ernstoff M.S., Hamid O., Lawrence D., McDermott D., Puzanov I., Wolchok J.D., Clark J.I., Sznol M., Logan T.F. (2012). Ipilimumab in patients with melanoma and brain metastases: An open-label, phase 2 trial. Lancet Oncol..

[87] A Tawbi H., A Forsyth P., Hodi F.S., Lao C.D., Moschos S.J., Hamid O., Atkins M.B., Lewis K., Thomas R.P., A Glaspy J. (2021). Safety and efficacy of the combination of nivolumab plus ipilimumab in patients with melanoma and asymptomatic or symptomatic brain metastases (CheckMate 204). Neuro-Oncol..

[88] Long G.V., Atkinson V., Lo S., Sandhu S., Guminski A.D., Brown M.P., Wilmott J.S., Edwards J., Gonzalez M., Scolyer R.A. (2018). Combination nivolumab and ipilimumab or nivolumab alone in melanoma brain metastases: A multicentre randomised phase 2 study. Lancet Oncol..

[89] A Tawbi H., A Forsyth P., Hodi F.S., Algazi A.P., Hamid O., Lao C.D., Moschos S.J., Atkins M.B., Lewis K., A Postow M. (2021). Long-term outcomes of patients with active melanoma brain metastases treated with combination nivolumab plus ipilimumab (CheckMate 204): Final results of an open-label, multicentre, phase 2 study. Lancet Oncol..

[90] Di Giacomo A.M., A Ascierto P., Pilla L., Santinami M., Ferrucci P.F., Giannarelli D., Marasco A., Rivoltini L., Simeone E., Nicoletti S.V. (2012). Ipilimumab and fotemustine in patients with advanced melanoma (NIBIT-M1): An open-label, single-arm phase 2 trial. Lancet Oncol..

[91] Di Giacomo A.M., Chiarion-Sileni V., Del Vecchio M., Ferrucci P.F., Guida M., Quaglino P., Guidoboni M., Marchetti P., Cutaia O., Amato G. (2021). Primary Analysis and 4-Year Follow-Up of the Phase III NIBIT-M2 Trial in Melanoma Patients With Brain Metastases. Clin. Cancer Res..

[92] Goldberg S.B., Gettinger S.N., Mahajan A., Chiang A.C., Herbst R.S., Sznol M., Tsiouris A.J., Cohen J., Vortmeyer A., Jilaveanu L. (2016). Pembrolizumab for patients with melanoma or non-small-cell lung cancer and untreated brain metastases: Early analysis of a non-randomised, open-label, phase 2 trial. Lancet Oncol..

[93] Kluger H.M., Chiang V., Mahajan A., Zito C.R., Sznol M., Tran T., Weiss S.A., Cohen J.V., Yu J., Hegde U. (2018). Long-Term Survival of Patients With Melanoma With Active Brain Metastases Treated With Pembrolizumab on a Phase II Trial. J. Clin. Oncol..

[94] Ascierto P.A., Del Vecchio M., Robert C., Mackiewicz A., Chiarion-Sileni V., Arance A., Lebbé C., Bastholt L., Hamid O., Rutkowski P. (2017). Ipilimumab 10 mg/kg versus ipilimumab 3 mg/kg in patients with unresectable or metastatic melanoma: A randomised, double-blind, multicentre, phase 3 trial. Lancet Oncol..

[95] Ascierto P.A., Del Vecchio M., Mackiewicz A., Robert C., Chiarion-Sileni V., Arance A., Lebbé C., Svane I.M., McNeil C., Rutkowski P. (2020). Overall survival at 5 years of follow-up in a phase III trial comparing ipilimumab 10 mg/kg with 3 mg/kg in patients with advanced melanoma. J. Immunother. Cancer.

[96] Pelster M.S., Amaria R.N. (2019). Combined targeted therapy and immunotherapy in melanoma: A review of the impact on the tumor microenvironment and outcomes of early clinical trials. Ther. Adv. Med Oncol..

[97] Tsao M., Xu W., Sahgal A. (2012). A meta-analysis evaluating stereotactic radiosurgery, whole-brain radiotherapy, or both for patients presenting with a limited number of brain metastases. Cancer.

[98] Tallet A.V., Azria D., Barlesi F., Spano J.-P., Carpentier A.F., Gonçalves A., Metellus P. (2012). Neurocognitive function impairment after whole brain radiotherapy for brain metastases: Actual assessment. Radiat. Oncol..

[99] Krummel D.A.P., Nasti T.H., Izar B., Press R.H., Xu M., Lowder L., Kallay L., Rupji M., Rosen H., Su J. (2020). Impact of Sequencing Radiation Therapy and Immune Checkpoint Inhibitors in the Treatment of Melanoma Brain Metastases. Int. J. Radiat. Oncol. Biol. Phys..

[100] Weil R.J., Palmieri D.C., Bronder J.L., Stark A.M., Steeg P.S. (2005). Breast Cancer Metastasis to the Central Nervous System. Am. J. Pathol..

[101] Sperduto P.W., Kased N., Roberge D., Chao S.T., Shanley R., Luo X., Sneed P.K., Suh J., Weil R.J., Jensen A.W. (2013). The effect of tumor subtype on the time from primary diagnosis to development of brain metastases and survival in patients with breast cancer. J. Neuro-Oncol..

[102] Schmid P., Adams S., Rugo H.S., Schneeweiss A., Barrios C.H., Iwata H., Diéras V., Hegg R., Im S.-A., Shaw Wright G. (2018). Atezolizumab and Nab-Paclitaxel in Advanced Triple-Negative Breast Cancer. N. Engl. J. Med..

[103] Cortes J., Cescon D.W., Rugo H.S., Nowecki Z., Im S.-A., Yusof M.M., Gallardo C., Lipatov O., Barrios C.H., Holgado E. (2020). Pembrolizumab plus chemotherapy versus placebo plus chemotherapy for previously untreated locally recurrent inoperable or metastatic triple-negative breast cancer (KEYNOTE-355): A randomised, placebo-controlled, double-blind, phase 3 clinical trial. Lancet.

[104] Berghoff A.S., Fuchs E., Ricken G., Mlecnik B., Bindea G., Spanberger T., Hackl M., Widhalm G., Dieckmann K., Prayer D. (2016). Density of tumor-infiltrating lymphocytes correlates with extent of brain edema and overall survival time in patients with brain metastases. Oncoimmunology.

[105] Waqar S.N., Samson P.P., Robinson C.G., Bradley J., Devarakonda S., Du L., Govindan R., Gao F., Puri V., Morgensztern D. (2018). Non–small-cell Lung Cancer With Brain Metastasis at Presentation. Clin. Lung Cancer.

[106] Shi A.A., Digumarthy S.R., Temel J.S., Halpern E.F., Kuester L.B., Aquino S.L. (2006). Does Initial Staging or Tumor Histology Better Identify Asymptomatic Brain Metastases in Patients with Non–small Cell Lung Cancer?. J. Thorac. Oncol..

[107] Sorensen J.B., Hansen H.H., Hansen M., Dombernowsky P. (1988). Brain Metastases in Adenocarcinoma of the Lung: Frequency, Risk Groups, and Prognosis. J. Clin. Oncol..

[108] Kelly K., A Bunn P. (1998). Is it time to reevaluate our approach to the treatment of brain metastases in patients with non-small cell lung cancer?. Lung Cancer.

[109] Patil T., Smith D.E., Bunn P.A., Aisner D.L., Le A.T., Hancock M., Purcell W.T., Bowles D.W., Camidge D.R., Doebele R.C. (2018). The Incidence of Brain Metastases in Stage IV ROS1-Rearranged Non–Small Cell Lung Cancer and Rate of Central Nervous System Progression on Crizotinib. J. Thorac. Oncol..

[110] Rangachari D., Yamaguchi N., VanderLaan P.A., Folch E., Mahadevan A., Floyd S.R., Uhlmann E.J., Wong E.T., Dahlberg S.E., Huberman M.S. (2015). Brain metastases in patients with EGFR -mutated or ALK -rearranged non-small-cell lung cancers. Lung Cancer.

[111] Peters S., Camidge D.R., Shaw A.T., Gadgeel S., Ahn J.S., Kim D.W., Ou S.H.I., Pérol M., Dziadziuszko R., Rosell R. (2017). Alectinib versus Crizotinib in Untreated ALK-Positive Non–Small-Cell Lung Cancer. N. Engl. J. Med..

[112] Reungwetwattana T., Nakagawa K., Cho B.C., Cobo M., Cho E.K., Bertolini A., Bohnet S., Zhou C., Lee K.H., Nogami N. (2018). CNS Response to Osimertinib Versus Standard Epidermal Growth Factor Receptor Tyrosine Kinase Inhibitors in Patients With Untreated *EGFR*-Mutated Advanced Non–Small-Cell Lung Cancer. J. Clin. Oncol..

[113] Urbanska E.M., Santoni-Rugiu E., Melchior L.C., Carlsen J.F., Sørensen J.B. (2020). Intracranial Response of ALK+ Non-Small-cell Lung Cancer to Second-line Dose-escalated Brigatinib After Alectinib Discontinuation Due to Drug-induced Hepatitis and Relapse After Whole Brain Radiotherapy Followed by Stereotactic Radiosurgery. Clin. Lung Cancer.

[114] Lin J.J., Jiang G.Y., Joshipura N., Ackil J., Digumarthy S.R., Rincon S.P., Yeap B.Y., Gainor J.F., Shaw A.T. (2019). Efficacy of Alectinib in Patients with ALK-Positive NSCLC and Symptomatic or Large CNS Metastases. J. Thorac. Oncol..

[115] Facchinetti F., Levy A., Ammari S., Naltet C., Lavaud P., Aldea M., Vasseur D., Planchard D., Besse B. (2021). Meningeal “Lazarus Response” to Lorlatinib in a ROS1-Positive NSCLC Patient Progressing to Entrectinib. Cancer Manag. Res..

[116] Hochmair M., Weinlinger C., Prosch H. (2019). Intracranial remission with brigatinib rechallenge as fifth-line ALK inhibition therapy in a lung cancer patient. Anti-Cancer Drugs.

[117] Koba T., Kijima T., Takimoto T., Hirata H., Naito Y., Hamaguchi M., Otsuka T., Kuroyama M., Nagatomo I., Takeda Y. (2017). Rapid intracranial response to osimertinib, without radiotherapy, in nonsmall cell lung cancer patients harboring the EGFR T790M mutation two case reports. Medicine.

[118] Molinier O., Besse B., Barlesi F., Audigier-Valette C., Friard S., Monnet I., Jeannin G., Mazières J., Cadranel J., Hureaux J. (2022). IFCT-1502 CLINIVO: Real-world evidence of long-term survival with nivolumab in a nationwide cohort of patients with advanced non-small-cell lung cancer. ESMO Open.

[119] Mansfield A.S., Herbst R.S., de Castro G., Hui R., Peled N., Kim D.-W., Novello S., Satouchi M., Wu Y.-L., Garon E.B. (2021). Outcomes With Pembrolizumab Monotherapy in Patients With Programmed Death-Ligand 1–Positive NSCLC With Brain Metastases: Pooled Analysis of KEYNOTE-001, 010, 024, and 042. JTO Clin. Res. Rep..

[120] Chen H., Feng Y., Zhou Y., Tao Y., Tang L., Shi Y. (2022). Brain metastases and immune checkpoint inhibitors in non-small cell lung cancer: A systematic review and meta-analysis. Cancer Immunol. Immunother..

[121] Camidge D.R., Lee E.Q., Lin N.U., Margolin K., Ahluwalia M.S., Bendszus M., Chang S.M., Dancey J., E de Vries E.G., Harris G.J. (2018). Clinical trial design for systemic agents in patients with brain metastases from solid tumours: A guideline by the Response Assessment in Neuro-Oncology Brain Metastases working group. Lancet Oncol..

[122] Schoenmaekers J.J.A.O., Dursun S., Biesmans C., De Ruysscher D.K.M., Broen M.P.G., Remon J., Dingemans A.-M.C., Hendriks L.E.L. (2021). Dynamics of eligibility criteria for central nervous system metastases in non-small cell lung cancer randomized clinical trials over time: A systematic review. Crit. Rev. Oncol./Hematol..

[123] El Rassy E., Botticella A., Kattan J., Le Péchoux C., Besse B., Hendriks L. (2018). Non-small cell lung cancer brain metastases and the immune system: From brain metastases development to treatment. Cancer Treat. Rev..

[124] Vilariño N., Bruna J., Bosch-Barrera J., Valiente M., Nadal E. (2020). Immunotherapy in NSCLC patients with brain metastases. Understanding brain tumor microenvironment and dissecting outcomes from immune checkpoint blockade in the clinic. Cancer Treat. Rev..

[125] Crinò L., Bronte G., Bidoli P., Cravero P., Minenza E., Cortesi E., Garassino M.C., Proto C., Cappuzzo F., Grossi F. (2019). Nivolumab and brain metastases in patients with advanced non-squamous non-small cell lung cancer. Lung Cancer.

[126] Goldberg S.B., Schalper K.A., Gettinger S.N., Mahajan A., Herbst R.S., Chiang A.C., Lilenbaum R., Wilson F.H., Omay S.B., Yu J.B. (2020). Pembrolizumab for management of patients with NSCLC and brain metastases: Long-term results and biomarker analysis from a non-randomised, open-label, phase 2 trial. Lancet Oncol..

[127] Hellmann M.D., Paz-Ares L., Bernabe Caro R., Zurawski B., Kim S.-W., Carcereny Costa E., Park K., Alexandru A., Lupinacci L., de la Mora Jimenez E. (2019). Nivolumab plus Ipilimumab in Advanced Non–Small-Cell Lung Cancer. N. Engl. J. Med..

[128] Gandhi L., Rodríguez-Abreu D., Gadgeel S., Esteban E., Felip E., De Angelis F., Domine M., Clingan P., Hochmair M.J., Powell S.F. (2018). Pembrolizumab plus Chemotherapy in Metastatic Non–Small-Cell Lung Cancer. N. Engl. J. Med..

[129] Gadgeel S., Rodríguez-Abreu D., Speranza G., Esteban E., Felip E., Dómine M., Hui R., Hochmair M.J., Clingan P., Powell S.F. (2020). Updated Analysis From KEYNOTE-189: Pembrolizumab or Placebo Plus Pemetrexed and Platinum for Previously Untreated Metastatic Nonsquamous Non-Small-Cell Lung Cancer. J. Clin. Oncol..

[130] Rittmeyer A., Barlesi F., Waterkamp D., Park K., Ciardiello F., von Pawel J., Gadgeel S.M., Hida T., Kowalski D.M., Dols M.C. (2017). Atezolizumab versus docetaxel in patients with previously treated non-small-cell lung cancer (OAK): A phase 3, open-label, multicentre randomised controlled trial. Lancet.

[131] Brastianos P.K., Strickland M.R., Lee E.Q., Wang N., Cohen J.V., Chukwueke U., Forst D.A., Eichler A., Overmoyer B., Lin N.U. (2021). Phase II study of ipilimumab and nivolumab in leptomeningeal carcinomatosis. Nat. Commun..

[132] Brastianos P.K., Lee E.Q., Cohen J.V., Tolaney S.M., Lin N.U., Wang N., Chukwueke U., White M.D., Nayyar N., Kim A. (2020). Single-arm, open-label phase 2 trial of pembrolizumab in patients with leptomeningeal carcinomatosis. Nat. Med..

[133] Grossman S.A., Krabak M.J. (1999). Leptomeningeal Carcinomatsis. Cancer Treat Rev..

[134] Le Rhun E., Preusser M., Bent M.V.D., Andratschke N., Weller M. (2019). How we treat patients with leptomeningeal metastases. ESMO Open.

[135] Beauchesne P. (2010). Intrathecal chemotherapy for treatment of leptomeningeal dissemination of metastatic tumours. Lancet Oncol..

[136] Iorgulescu J.B., Gokhale P.C., Speranza M.C., Eschle B.K., Poitras M.J., Wilkens M.K., Soroko K.M., Chhoeu C., Knott A., Gao Y. (2021). Concurrent Dexamethasone Limits the Clinical Benefit of Immune Checkpoint Blockade in Glioblastoma. Clin. Cancer Res..

